# Surgical Management of Desmoid Tumors—Patient Selection, Timing, and Approach

**DOI:** 10.3390/curroncol32070408

**Published:** 2025-07-18

**Authors:** Catherine Sarre Lazcano, Alessandro Gronchi

**Affiliations:** 1 Division of Surgical Oncology, Department of Surgery, University of Toronto, Toronto, ON M5S 1A1, Canada; reynacatherine.sarrelazcano@uhn.ca; 2 Sarcoma Service, Department of Surgery, Fondazione IRCCS Istituto Nazionale dei Tumori, 20133 Milan, Italy

**Keywords:** desmoid tumor, aggressive fibromatosis, surgery, margins, functional outcomes

## Abstract

Desmoid tumors are rare, deep-seated myofibroblastic tumors with an unpredictable course, ranging from spontaneous regression to infiltrative growth and locally aggressive behavior, but without metastatic potential. Over the past few decades, advances in understanding their natural history, underlying molecular pathways, and patient care priorities have shifted the treatment paradigm from upfront surgical resection to initial active surveillance, with further treatment dictated by continuous disease progression or associated symptoms. However, there are still specific scenarios where surgery continues to play an important role in locoregional treatment and symptom control. This article will focus on current treatment strategies and surgical indications in adult patients with desmoid tumors, emphasizing patient selection, anatomic site-specific considerations, and surgical technique. Understanding the nuanced role of surgery within the growing treatment landscape is key for individualized patient care in a multidisciplinary setting to optimize quality of life and long-term outcomes.

## Simple Summary

Desmoid tumors are rare, non-metastatic tumors that form in connective tissues such as muscles, ligaments, or fibrous tissue. They can behave unpredictably—some remain stable or shrink on their own, while others grow aggressively and cause significant local issues without spreading to distant organs. Historically, surgery was the first-line treatment, but a better understanding of the disease has shifted the standard toward active surveillance as the initial approach. Treatment is now typically reserved for tumors that show continuous growth or cause symptoms. Despite this shift, surgery still plays an important role in select cases, particularly when tumors affect nearby structures or impair function. This article reviews current treatment strategies for adult patients with desmoid tumors, focusing on appropriate surgical indications, how tumor location influences decision-making, and key technical considerations. The goal is to clarify surgery’s role within an individualized, multidisciplinary care plan aimed at optimizing outcomes and preserving quality of life.

## 1. Introduction

Desmoid tumors (DTs) are rare, deep-seated myofibroblastic neoplasms characterized by a monoclonal fibroblastic proliferation of intermediate malignant potential [1]. Their global incidence is estimated at 2–6 cases per million people per year [2,3], with a predilection for patients aged 20–44 years, a median age of presentation of 35–40 years, and a notable female predominance (~70%) [4]. DTs exhibit highly variable clinical courses, ranging from asymptomatic tumors with spontaneous regression to infiltrative growth and locally aggressive behavior, often causing significant symptom burden and quality of life impairment. While they lack metastatic potential, they can occasionally be multifocal [5]. Large size, involvement of adjacent structures, and local recurrence (LR) contribute importantly to morbidity and, in some cases, mortality, highlighting the need for individualized treatment strategies based on patient, tumor, and anatomical characteristics [6].

### 1.1. Mutational Drivers and Clinical Implications

DTs can present in two settings: (1) sporadic tumors (85%), driven by somatic mutations in the CTNNB1 gene encoding β-catenin, and (2) familial adenomatous polypomatosis (FAP)-related (10–15%), associated with germline mutations in the adenomatous polyposis coli (APC) gene (10–15%) [7]. Both mutations result in dysregulation of the Wnt signaling pathway, promoting overexpression of genes involved in proliferation and fibrosis [8].

The mutational profile impacts not only clinical presentation but also treatment decisions and prognosis. DTs develop in 10–15% of patients with FAP (an association known as Gardner syndrome), rising to 25% when there is a family history of DTs [9]. While only 5% of sporadic DTs are intra-abdominal (IA), 80% of FAP-related DTs arise in the abdominal cavity, with 10–15% in the abdominal wall (AW) and 5–10% in extra-abdominal sites [10,11]. Surgical trauma is a significant risk factor for FAP-related DTs, with up to 80% occurring after prior surgical interventions (namely prophylactic colectomy at an early age), at a median onset of 22 months post-surgery [10]. Finally, while DT-related mortality is negligible in sporadic DTs [12], it can reach 20% in FAP-related DTs [13].

The most common location of sporadic DTs is the AW (30–55%), followed by extremity (15–45%), chest wall (12–27%), head and neck (4–10%), and IA (2–5%) [14,15,16]. Morbidity is mainly associated with mass effect, causing pain, altered cosmesis, or restricted mobility. Symptoms may also be related to compression or infiltration of surrounding structures [17]. Location may increase the risk of life-threatening complications, such as in head and neck DTs.

### 1.2. Paradigm Shift: Active Surveillance as Frontline Approach

Historically, DTs were treated with upfront surgical resection and wide clinical margins. However, this approach often resulted in high LR rates—ranging from 24 to 77% for extremity DTs and 57–86% for IA disease, especially in the context of the Gardner syndrome—along with suboptimal functional outcomes due to postoperative morbidity and the infiltrative nature of the disease [3]. Increasing evidence of their unpredictable clinical course and potential for stabilization or even spontaneous regression led to a shift away from surgery as first-line treatment. By the early 2000s, active surveillance (AS) emerged as the preferred initial approach for both primary and recurrent tumors [18]. AS allows for observation of tumor biology, potentially sparing patients from treatment-related morbidity, while allowing for therapeutic intervention in case of significant progression or symptom development.

AS has been evaluated in three European prospective studies, including patients with primary sporadic DTs [14,16,19]. Overall, nearly 60% of patients had spontaneous regression, 25–35% after initial presentation, and 23–31% after initial progression. It is worth highlighting this latter group, as they may avoid the morbidity of active treatment if regression is documented during the initial years following presentation and even initial progression. Approximately 30% of patients required active treatment due to AS failure, characterized by continued tumor progression or worsening symptoms. Surgical resection was performed in only 1–12% of cases.

While these studies demonstrate that AS is a safe and effective strategy for identifying patients who require treatment, it is worth noting that they included only a small number of patients with IA and head and neck DTs. No clear risk factors for AS failure were identified, though larger tumor size (>5 cm), extremity location, S45F β-catenin mutation, and pregnancy were noted as potential predictors of progression. Additionally, no standardized criteria exist for defining the threshold of dimensional increase or symptom severity that warrants consideration of active treatment. Nevertheless, this evidence strongly supports delaying intervention until sustained progression or significant symptom burden occurs. When considering active treatment, the risks and benefits must be carefully weighed, considering potential morbidity, the likelihood of local complications, and the risk of progression or recurrence.

Major international guidelines on DT management include the Global Consensus from the Desmoid Tumor Working Group (DTWG) [20], the National Comprehensive Cancer Network (NCCN) guidelines [21] and the European Society of Medical Oncology (ESMO) Practice Guidelines [22]. All emphasize the importance of accurate pathology diagnosis and initial observation to assess progression while carefully considering symptom burden and anatomic location before initiating active treatment.

### 1.3. Optimizing Outcomes: The Importance of Multidisciplinary Management in Referral Centers

Given the rarity of DTs, their complex natural history, rapidly evolving treatment landscape, and highly nuanced management, early patient referral to specialized sarcoma centers with the necessary expertise and resources is crucial for ensuring optimal care. Key factors influencing treatment approach are summarized in Table 1. Active treatment is recommended when there is progression after initial AS. This is defined by guidelines [20,21] as any of the following:

(a) Persistent tumor growth documented on follow-up imaging, defined as an increase in tumor size across three or more follow-up visits, or 24 months [18].
(b) Impairment or threat to life, function or quality of life.
(c) Worsening or progressive symptoms.

The recommendations for first-line treatment modality are mainly determined by anatomic location and, in the context of IA DTs, if they are sporadic or FAP-related [20]. Other factors such as the proportion and speed of growth, symptom severity, potential morbidity, and expected effectiveness of the therapeutic option must also be considered [23].

Primary treatment options may include systemic therapies, such as low-dose chemotherapy, conventional chemotherapy [24], tyrosine kinase inhibitors [25,26], or gamma-secretase inhibitors [27]. Agent selection should account for safety profile, estimated treatment duration, and drug availability. In patients with a local disease such as desmoid, systemic therapy may be considered disproportionate due to potential toxicity—particularly in the predominantly young female patient population, where preservation of ovarian function and reproductive concerns are significant. Additionally, treatment duration is often undefined, and access to targeted agents may be limited by cost or availability. These challenges may support the consideration of surgery as a viable option when appropriate. Conversely, systemic therapies may represent a valuable option for symptom control and quality-of-life improvement in patients for whom surgical morbidity or recurrence risk is deemed high. This is particularly true in mesenteric or head and neck DTs, where the disease location, extent, and natural history may prohibit other elective treatment options [20].

Locoregional therapies are a growing area of research, representing a minimally invasive option that may offer effective symptom relief and preserve function. Image-guided interventional radiology options include thermal and chemical ablation, arterial chemoembolization [28], high-intensity focused ultrasound, and microwave and radiofrequency ablation [29]. The efficacy and safety of cryoablation have been reported in several single-center studies [30], with phase 2 results already available [31] and ongoing recruitment in a prospective randomized control trial. Disadvantages may include decreased effectiveness with larger tumors, the potential need for repeated treatments, damage to adjacent neurovascular structures, or residual mass effect [32].

Definitive, moderate-dose radiotherapy (RT) may have a role as a secondary treatment option in non-mesenteric DTs, such as head and neck, extremities, girdles, chest, or AW [33]. RT can be a valuable therapy when systemic treatments are ineffective and surgical resection is challenging given anatomic and technical constraints [34]. There is no current role for preoperative or adjuvant radiotherapy, regardless of margin status.

Isolated limb perfusion (ILP) has been reported as a secondary treatment option in patients with extremity DTs as a stand-alone treatment, in combination with other regional therapies, or as a bridge to surgical resection [35]. Current indications include progressive disease (PD) after primary active treatment options have been exhausted in patients with severe impact on quality of life and where surgical morbidity is prohibitive [20].

When selecting the optimal approach for each patient, the benefits and risks of any active treatment modality should be carefully weighed in a multidisciplinary discussion setting, including medical oncology, interventional radiology, and surgical oncology. This article presents a proposed framework outlining current surgical treatment indications of patients with DTs based on their anatomic location and presentation.

## 2. The Role of Surgical Management in Desmoid Tumors

**Figure 1:**
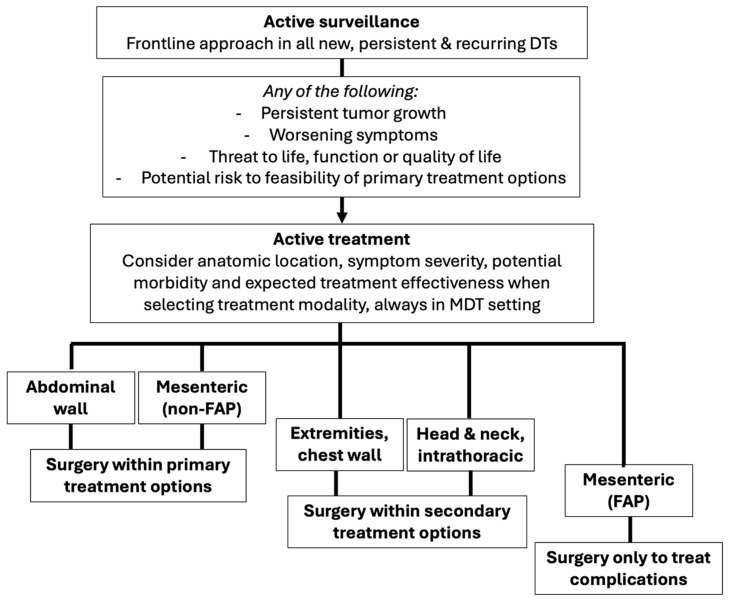
Role of surgical management in desmoid tumors according to anatomic location. Adapted from: Kasper B et al., JAMA Oncology. 2024;10(8):1121-1128 [20]. DTs: desmoid tumors; FAP: familial adenomatous polypomatosis; MDT: multidisciplinary team.

While only a small percentage of patients with DTs will require surgical treatment, it remains a relevant primary or secondary treatment option, depending on the clinical scenario. Patient selection, timing, and surgical approach should be carefully planned to optimize both function and quality of life, always in the setting of multidisciplinary management (Figure 1). Factors such as perioperative risks and morbidity, expected functional outcomes, pre-existing or postoperative chronic pain, and risk of LR must be evaluated when determining whether surgery is an appropriate treatment option. Surgical challenges, including type and extent of reconstruction, involvement of critical structures, and expected functional impact, must also be considered.

When indicated, general principles for surgical resection of desmoid tumors include the following:

- **Who?** Anatomic location and FAP status are the main determinants of surgical eligibility, as they directly influence the risk of postoperative complications and long-term outcomes. In general, sporadic AW DTs derive the greatest benefit from resection, followed by sporadic mesenteric DTs.
- **When?** Surgical resection must only be considered in case of sustained documented progression as defined above, given that approximately two-thirds of patients may experience spontaneous regression. All cases must be discussed in a multidisciplinary setting to ensure consideration of all available treatment options.
- **How?** Resection should aim to achieve complete macroscopic excision with minimal to narrow margins while preserving function. Since LR rates are not significantly impacted by R0 vs. R1 margins, wider resections offer no added benefit and may increase the need for soft tissue reconstruction, associated surgical morbidity, and functional impairment [36].

### 2.1. Risk of Recurrence After Surgery

Whenever active treatment is indicated and surgery is being considered, the risk of disease recurrence should be carefully assessed. Tumor site (extremity, chest wall, and head and neck DTs), larger tumor size, and younger patient age have been identified as risk factors for LR, supporting a multimodality treatment approach in these patients. A validated prognostic nomogram for predicting LR is available for aiding clinical decision-making [37]. Patients presenting with recurrent disease after initial resection are also at increased risk of re-recurrence [38] and should undergo AS as an initial strategy. If active treatment is indicated in these cases, non-surgical treatment options should be favored.

### 2.2. Patient-Related Outcomes

Although DTs lack metastatic potential, they can cause debilitating symptoms due to their locally infiltrating behavior and tendency to recur. Their unique disease course requires that the treating specialists have in-depth knowledge of the potential symptoms and their impact on quality of life (QoL) [39]. Pain is reported in up to 36% of patients at diagnosis, may worsen even in the absence of radiologic disease progression, and is associated with decreased event-free survival (including recurrence, progression, or death) [40]. In addition to physical symptoms, DTs can exert a significant psychosocial toll. DT-specific QoL assessment tools are essential for accurately tracking symptom burden, guiding decisions on initiating active treatment, evaluating therapeutic effectiveness, and monitoring patient functional status [41,42].

## 3. Abdominal Wall DTs

**Figure 2:**
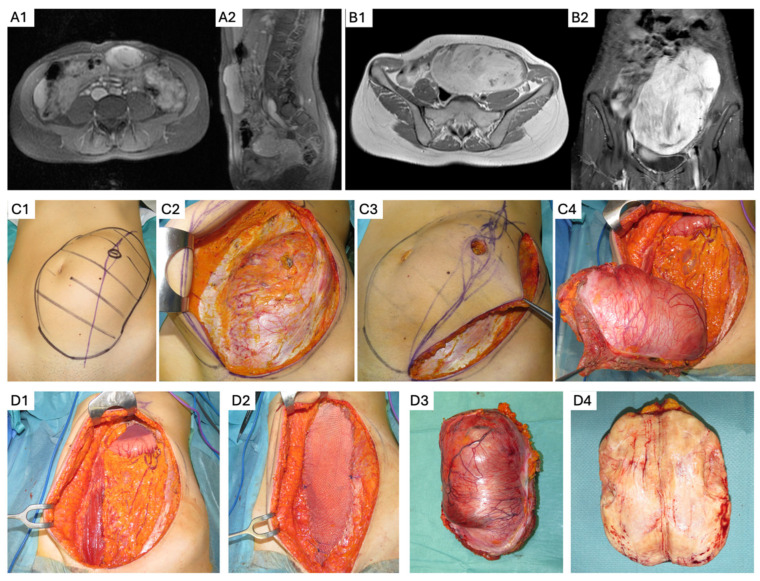
Surgical management of abdominal wall desmoid tumor. (**A1**,**A2**) Axial and sagittal T1 MRI of a sporadic lower abdominal wall desmoid in a 30-year-old female patient. (**B1**,**B2**) She had progressive growth and increasing symptoms, remaining on active surveillance for 2 years. (**C1**–**C4**) She underwent myofascial resection, including the biopsy tract, with narrow clinical margins and mesh reconstruction (**D1**,**D2**), with no complications. Pathology showed negative margins (**D3**,**D4**), and she underwent surveillance. All surgical images of this manuscript were retrospectively collected from routine clinical procedures and have not been published before. The use of these images is in accordance with the Declaration of Helsinki. The patient consent is waived because of the retrospective analysis, and all patient identifiers have been removed.

The AW is the most common anatomic location for sporadic DTs, particularly among female patients of reproductive age [43]. When active treatment is indicated, primary options also include systemic therapies and local ablative techniques [20]. Sporadic AW DTs are often considered the most favorable setting for surgical treatment (Figure 2), given the patient population characteristics and disease natural history [37]. The median 5-year LR rate is low (8%), with minimal morbidity and no mortality [44].

Key recommendations for surgical resection of AW DTs include the following:

- Whenever possible, overlying skin and subcutaneous adipose tissue should be preserved to decrease the need for cutaneous flap reconstruction. A fascia-preserving technique has been described by Nishida et al., who performed a marginal, macroscopically complete resection that reduces or eliminates the need for fascial resection and reconstruction, with a reported LR rate of 6.7% [45]. However, definitive data are lacking, and full-thickness AW resection remains the conventional approach when it can be performed with an acceptable functional impact.
- If myofascial AW resection is required, reconstruction techniques should be selected based on the extent of the fascial defect, patients’ clinical characteristics, and risk of incisional hernia. When significant soft tissue or fascial resection is anticipated, multidisciplinary surgical planning—including collaboration with AW reconstruction or plastic surgery specialists—should be pursued to optimize functional and cosmetic outcomes. Expected postoperative results should be discussed preoperatively to align with patient expectations and priorities.
- For smaller fascial defects, primary fascial closure with relaxing incisions may be sufficient and can be reinforced with an onlay or sublay mesh if needed [46]. In cases of larger myofascial defects, bridging mesh reconstruction may be required. The choice of mesh composition should be tailored to the risk of surgical site infection, wound contamination, and contact with IA contents [47].
- In patients with potential future pregnancies, alternatives to mesh reconstruction should be considered to maximize future AW compliance. These strategies may include preoperative botulinum toxin A injection [48] to facilitate a tension-free, primary midline fascial closure with a reinforced tension-line suture technique [49]. If primary fascial closure is achieved, but there is a high risk of incisional hernia or non-midline closure, reinforcement with an onlay or sublay, slow-reabsorbing synthetic mesh may be appropriate [43,50].
- When myofascial tissue reconstruction is required, advanced techniques such as component separation or autologous reconstruction with pedicled or free flaps may be indicated for full-thickness defects [51]. Alternatively, bridging mesh reconstruction with slow-reabsorbing synthetic mesh can be considered. Even in patients with permanent synthetic mesh reconstruction, future pregnancy is not contraindicated, though it carries a higher risk of pain during the third trimester [52] and chronic pain [53]. Close obstetric monitoring is recommended to ensure fetal and maternal well-being.

## 4. Desmoids and Pregnancy

DTs are present 2–3 times more frequently in female patients of child-bearing age, with a median age at diagnosis of 35–40 years. Given this epidemiologic profile, pregnancy and fertility are key concerns in this patient population. Pregnancy-associated DTs are recognized as a distinct subtype, most often arising in the AW, either during or within 2 years of pregnancy [54]. Interestingly, hormonal exposure (oral contraceptives or estrogen replacement) has not been shown to influence the incidence or outcomes of DTs [55].

AS is currently recommended as the first-line treatment strategy for pregnancy-associated DTs, with a progression rate (33%) and median time to progression (18 months) similar to DTs in general [54]. While some studies have reported an association between pregnancy and LR or progression [55], most cases do not require active treatment or show spontaneous regression [56].

When active treatment is indicated (less than 6% of cases), recommendations for AW DTs can be followed. Pregnancy itself does not constitute an indication for active treatment, and there is no recommendation to delay pregnancy in patients with in situ or resected DTs [57]. Although close monitoring during pregnancy is advised, there is no evidence of increased obstetric risk in these patients [58]. Risk factors for progression include multiple pregnancies, increasing maternal age, and disease progression during prior pregnancies. In summary, pregnancy-associated DTs have favorable outcomes and can follow treatment recommendations for DTs in general, particularly when diagnosed and/or treated prior to pregnancy.

## 5. Sporadic Intra-Abdominal Desmoids

While fewer than 5% of all sporadic DTs are IA, the majority of IA DTs are sporadic (74–87%) [59]. They most commonly present within the mesentery but may also arise in the retroperitoneum or pelvis. Given their marked differences in disease course and outcomes, a thorough personal and family history, clinical examination, lower gastrointestinal endoscopy, and assessment of β-catenin status on core needle biopsy [60] should be performed at diagnosis to guide optimal patient management, particularly when considering surgical treatment [61]. Primary treatment options in this patient population also include medical therapies. Resect ability, expected morbidity, and the potential for complications in case of further progression or residual disease must be carefully evaluated when selecting treatment, given their proximity to critical structures.

**Figure 3:**
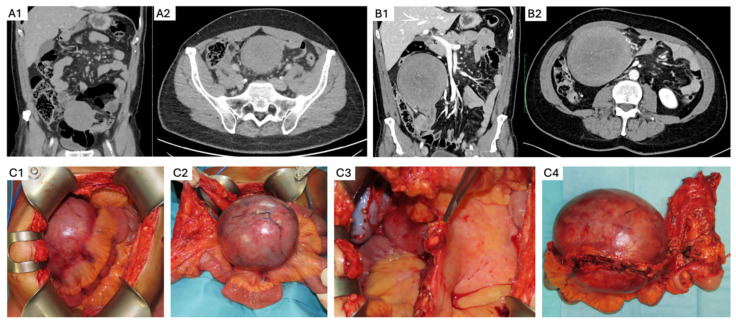
Surgical management of intraabdominal sporadic desmoid tumor. (**A1**,**A2**) A 59-year-old male patient presented with a palpable abdominal mass. Biopsy showed a DT, β-catenin-mutated. He underwent active surveillance. (**B1**,**B2**) A total of 2 months later, he presented with duodenal obstruction. CT showed rapid progression of the mesenteric DT. (**C1**–**C4**) He underwent an uncomplicated mesenteric and small bowel-sparing resection (**C1**,**C2**) with minimal margins (**C3**,**C4**) and radiologic surveillance, with no recurrence.

Surgery for sporadic IA DTs may be considered as an option in case of PD after AS in the following settings:

- **Elective resection as primary treatment.** In patients with resectable primary disease and low anticipated morbidity, complete macroscopic clearance can be achieved (Figure 3) with favorable operative outcomes in specialist centers [62]. When assessing resectability, key considerations include mesenteric vasculature involvement, anticipated length of small bowel and/or colonic resection, risk of short gut syndrome, and additional visceral involvement. The potential for future tumor growth and associated complications must also be carefully weighed [20,59]. In case of prior incomplete resection and evidence of residual disease, AS is recommended due to the possibility of an indolent course [63].
- **PD or intolerance to systemic therapy.** Surgery may be considered as an alternative treatment option for patients who develop treatment-limiting toxicity during systemic therapy, or in the case of PD, provided the tumor remains resectable with acceptable morbidity. This decision must be made in a multidisciplinary setting, considering the rate of progression, required extent of resection, current symptom burden, potential quality of life improvement, and availability of additional medical therapy options [34].
- **Surgical management of complications.** Sporadic IA DTs may be associated with complications such as bowel obstruction, perforation, bleeding, or intestinal ischemia in up to 10% of cases [64]. These complications can arise at initial presentation, during AS, or active treatment. Treatment should be guided by the patient’s clinical condition, type and severity of the complication, extent and resectability of the underlying disease, and compounded surgical morbidity. Management options include:
  ○ **Surgical treatment of complications with synchronous tumor resection,** if complete macroscopic resection is feasible and potential morbidity—such as the anticipated length of remnant bowel—is acceptable. This approach should only be considered if the diagnosis of a sporadic IA DT has been established and FAP has been excluded prior to the complication.
  ○ **Surgical management of complications without tumor resection,** aimed at stabilizing the patient clinically. This allows for further assessment of remnant bowel, postoperative symptom burden, functional status, extent of disease, and exclusion of FAP, all of which are critical for guiding primary treatment selection.

## 6. FAP-Associated Intraabdominal Desmoids

IA DTs account for approximately 75% of all FAP-associated DTs, arising most commonly in the mesentery. Risk factors for developing DTs in the context of FAP include prior abdominal surgery (namely open vs. laparoscopic colectomy) [65], female sex, family history of DTs, and specific APC gene mutations [66]. Compared with their sporadic counterparts, FAP-associated IA DTs are characterized by a more infiltrative and locally aggressive course, with historically higher rates of morbidity (22–60%), LR (65–88%), and disease-specific mortality (15–36%) [43,59]. In patients with a confirmed APC mutation, a mesenteric mass is highly suggestive of a DT, and multidisciplinary management including sarcoma and gastrointestinal oncology experts is recommended. Caution has been advised when considering biopsying FAP-associated IA DTs, given the risk of life-threatening complications [67].

**Figure 4:**
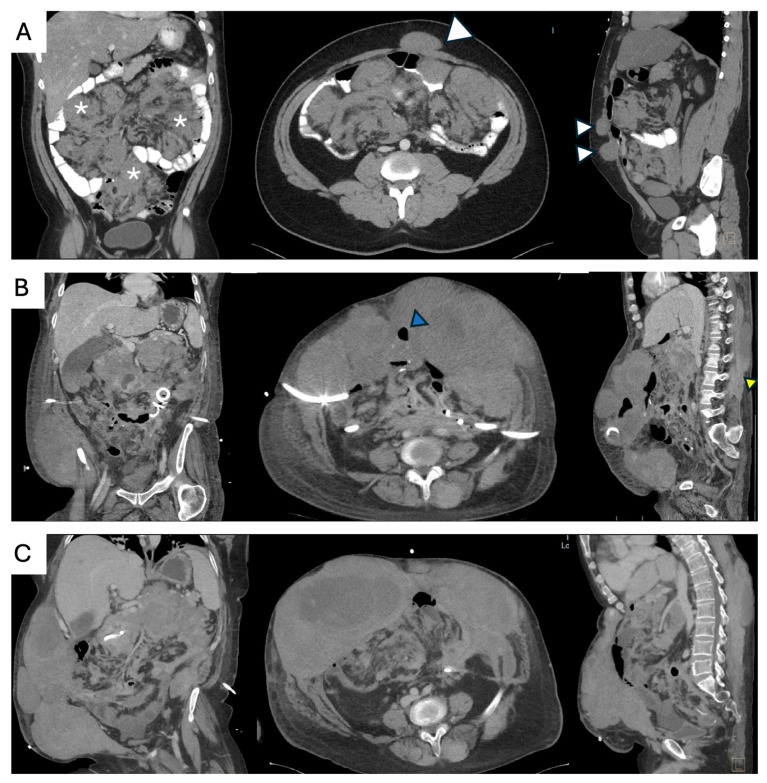
FAP-associated intraabdominal desmoid tumor. A 37-year-old male patient with FAP and a history of a total proctocolectomy with a bloc resection of a mesenteric desmoid. (**A**) He developed rapid multifocal mesenteric (asterisks) and abdominal wall (white arrows) recurrence 1 year later and was started on systemic treatment. (**B**) Development of progressive multifocal disease with fistulization (blue arrow) and abdominal sepsis, as well as new extra-abdominal disease (yellow arrow). (**C**) Continued disease progression with associated intestinal failure, requiring total parenteral nutrition, suppressive antibiotic therapy, and long-standing percutaneous drains.

Modern retrospective case series have reported on surgical management of FAP-associated IA DTs managed at specialist referral centers [64,66]. Emergency surgical intervention due to complications ranged between 16 and 56%, with palliative procedures performed in 12 and 75% of cases (Figure 4). No postoperative mortality was reported in this highly selected patient population. Macroscopic complete resection was achieved in 25 and 50% of cases, with a reported 5-year LR of 37% [66].Intestinal and modified multivisceral transplantation have been reported as salvage options in 14 patients with FAP-associated IA DTs with intestinal failure, with a 71% postoperative complication rate, 90% nutritional autonomy, 3-year LR of 42%, and 79% estimated 10-year survival [68].

The current role of surgery in FAP-associated IA DTs is reserved for the management of impending or life-threatening complications, such as bowel obstruction, perforation, bleeding, or intestinal ischemia. In these settings, the main objective of surgical intervention is symptom relief and control of the underlying complication, rather than complete tumor resection. This conservative surgical approach is recommended due to the potential for spontaneous regression or an indolent disease course, the high risk of procedure-related morbidity and disease-specific mortality, the potential for short bowel syndrome and intestinal failure, the infiltrative and fistulizing nature of these tumors, and the high rates of local recurrence following resection [69]. The extent of surgical intervention is dictated by the patient’s clinical condition and disease burden and must be tailored to each case.

## 7. Unfavorable Locations

**Figure 5:**
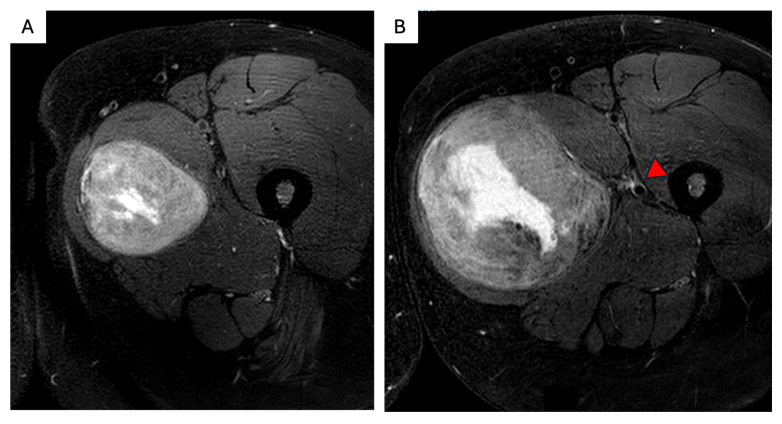
Surgical management of lower extremity desmoid tumor. (**A**) 46-year-old female patient with a desmoid tumor of the left medial thigh, on active surveillance for the past 4 years. (**B**) Progressive growth over 18 months continued to progress over sorafenib. Case discussed at MDT, deemed too large for cryoablation (>10 cm) and close to neurovascular structures (red arrow). She underwent an uncomplicated complete macroscopic resection and surveillance afterwards.

Surgical resection is generally not recommended for extra-abdominal DTs due to the considerable postoperative morbidity and LR rates, which are comparable to progression rates observed during AS [70]. This category includes DTs of the extremities, shoulder and pelvic girdles, chest wall, intrathoracic, and head and neck (H&N). Surgery may be considered when all available first-line treatment options have been exhausted (Figure 5) or when disease progression would likely involve critical structures [71]. At present, available data do not provide a sufficient basis to recommend a specific sequence of first-line active treatment modalities or to favor one over another, being highly dependent on patient and tumor characteristics. Any decisions regarding surgical intervention must be discussed in a multidisciplinary expert setting. In such cases, the potential functional impact, expected surgical morbidity, and risk of recurrence must be carefully balanced against the involvement of critical structures and the anticipated morbidity associated with tumor progression [20]. DTs arising in the shoulder girdle exhibit the highest propensity for progression [70]. While these considerations are relevant across all DT locations, they hold particularly true in these anatomically complex regions.

**Figure 6:**
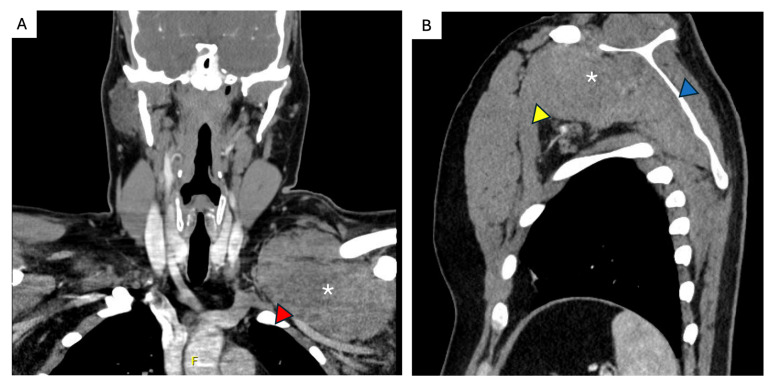
Shoulder girdle desmoid tumor. (**A**) A 27-year-old male patient with a history of left glenohumeral traumatic dislocation 3 years prior was referred for a 12-month history of a sporadic DT of the left shoulder girdle (asterisk), stable in size but with worsening pain and restricted range of motion. Coronal CT shows compression of the axillary vessels (red arrow). (**B**) Sagittal view shows an intimate relationship with neurovascular structures (yellow arrow) and loss of fat plane with the subscapularis muscle (blue arrow). He underwent first-line medical therapy, with symptomatic improvement.

When active treatment is pursued due to tumor-related pain, the potential benefit must be weighed against the risk of postoperative pain, which can be equally debilitating and significantly impact quality of life. Pain is more commonly reported in H&N and shoulder girdle DTs [40] (Figure 6). If surgery is considered, the extent of resection should be tailored to minimize morbidity and prioritize function; R0 should not be the primary goal. In case of residual disease or high LR risk, postoperative radiation can be considered to mitigate this risk, although it does not improve overall survival. The potential for late radiation-associated complications, including secondary malignancies, must be discussed preoperatively.

## 8. Follow up

The optimal follow-up frequency depends on factors such as anatomic location, treatment strategy, disease course, and associated symptoms. After surgical resection, the median time to LR ranges from 13 to 25 months [72,73], while the median time to progression or initiating active treatment after AS is between 6 and 20 months [74]. Therefore, more frequent imaging during the first 2 years (every 4–6 months) is recommended, which can be tailored based on patient symptoms and the morbidity of potential LR or progression [21]. The pattern of disease growth may not be constant, with periods of quiescence or accelerated growth [38].

## 9. Conclusions

Up to 60% of DTs will not require active treatment, and 30% will have spontaneous regression. When indicated, surgery may be considered in AW and mesenteric, non-FAP DTs after sustained progression on active surveillance and discussion in a multidisciplinary setting. Other primary treatment options, such as medical and locoregional therapies, should be considered according to patient characteristics, goals of treatment, and anatomic considerations. Surgery can be considered as a secondary treatment option in critical locations such as the head and neck, thorax, extremities, or girdles if there are no primary options available. The goal of surgical resection is to achieve a macroscopic complete resection with minimal margins, limiting postoperative morbidity, and preserving function. Surgical treatment in patients with FAP-related intra-abdominal DTs should only be performed in the setting of complications requiring emergency intervention. The landscape of treatment options for patients with DTs continues to evolve rapidly.

## Figures and Tables

**Table 1:** Key considerations in desmoid tumor approach and management.

Clinical Presentation	Treatment Strategies
Systems of care Multidisciplinary team in sarcoma referral center	Therapeutic goals Optimize tumor control and improve quality of life
Pathology diagnosis Expert sarcoma pathologist review Mutational testing and genotype characterization Sporadic vs. FAP-related desmoid	Active surveillance First-line approach in all new and recurrent DTs Decision on timing and management modality of active treatment if persistent tumor growth or worsening symptoms Optimal frequency, imaging modality and duration of follow up according to location, growth and symptoms
Tumor characteristics Anatomic location and extent of disease Compression or infiltration of surrounding structures Clinical course and behavior over time	Systemic treatment Agent selection: according to growth rate, severity of symptoms, patient characteristics and toxicity profile Optimal duration of treatment
Patient characteristics Age and functional status Personal priorities and goals of care Fertility and pregnancy planning	Locoregional therapies Cryoablation: symptomatic or growing DT refractory to medical treatment Radiation: lesions too large for cryoablation and unacceptable surgical morbidity Other therapies *
FAP-related desmoids Risk of intraabdominal complications and associated morbidity and mortality Genetic counseling; screening for associated neoplasms; surgical counseling regarding prophylactic colectomy	Surgical resection AS failure; favorable location, acceptable morbidity Emergency scenarios: bleeding, obstruction, perforation Potential for complications if further progression or residual disease
Symptom burden Risk or occurrence of life-threatening complications Functional impact and mobility Quality of life: cosmesis, chronic pain	Clinical trials New systemic therapies Locoregional treatment options Quality of life assessment and patient-related outcomes

AS: active surveillance; DT: desmoid tumor; FAP: familial adenomatous polypomatosis. * High-intensity focused ultrasound, isolated limb perfusion, thermal ablation techniques, and transarterial chemoembolization can be considered according to individual characteristics, multidisciplinary discussion, center capacity, and clinical trial availability. Based on recommendations from the Desmoid Tumor Working Group and National Comprehensive Cancer Network Guidelines.

## Data Availability

Not applicable.
